# “Cerebral small vessel disease and other influential factors of cognitive impairment in the middle-aged: a long-term observational cohort PURE-MIND study in Poland”

**DOI:** 10.1007/s11357-020-00271-4

**Published:** 2020-10-19

**Authors:** Dorota Szcześniak, Joanna Rymaszewska, Anna Zimny, Marek Sąsiadek, Katarzyna Połtyn-Zaradna, Eric E. Smith, Katarzyna Zatońska, Tomasz Zatoński, Sumathy Rangarajan, Salim Yusuf, Andrzej Szuba

**Affiliations:** 1 Department of Psychiatry, Wroclaw Medical University, Pasteura 10, 50-367 Wroclaw, Poland; 2 Department of General and Interventional Radiology and Neuroradiology, Wroclaw Medical University, Borowska 213, 50-556 Wroclaw, Poland; 3 Department of Social Medicine, Wroclaw Medical University, Bujwida 44, 50-345 Wroclaw, Poland; 4 Department of Clinical Sciences and Radiology, Hotchkiss Brain Institute, University of Calgary, Calgary, Canada; 5 Department and Clinic of Otolaryngology, Head and Neck Surgery, Wroclaw Medical University, Borowska 213, 50-556 Wroclaw, Poland; 6 Population Health Research Institute, Hamilton, ON Canada; 7 Department of Medicine, Faculty of Health Sciences, McMaster University, Hamilton, Ontario Canada; 8 Department of Angiology, Hypertension and Diabetology, Wroclaw Medical University, Borowska 213, 50-556 Wroclaw, Poland

**Keywords:** Cognitive impairment, Influential factors, Cerebral small vessel disease, Middle-aged population

## Abstract

A complex picture of factors influencing cognition is necessary to be drawn for a better understanding of the role of potentially modifiable factors in dementia. The aim was to assess the prevalence and determinants of cognitive impairment, including the role of cerebral small vessel disease (CSVD) in Polish middle-aged cohort. A comprehensive set of clinical (hypertension, coronary heart disease, diabetes mellitus, hyperlipidaemia, body mass index, smoking status, alcohol intake) and socio-demographic data was collected in the PURE study in years 2007–2016, which was the basis for detailed analysis of risk factors of cognitive impairments in years 2016–2018 in the PURE-MIND sub-study. Five hundred forty-seven subjects (age range 39–65, mean 56.2 ± 6.5) underwent neuropsychological assessment with Montreal Cognitive Assessment (MoCA), Trail Making Test (TMT) and Digit Symbol Substitution Test (DSST) followed by brain MRI. Mean MoCA score was 26.29 and 33% participants met criteria for mild cognitive impairment (MCI) (MoCA< 26). Seventy-three percent showed findings related to CSVD. Higher WMH burden and lacunar infarcts were associated with lower MoCA and DSST scores. Severe CSVD was associated with twofold incidence of MCI, and obesity increased its probability by 53% and hypertension by 37%. The likelihood of MCI was reduced in nonsmokers. One factor analysis showed the important role of lower level of education, older age, rural area of residence and hypertension. MCI and CSVD are highly prevalent in the middle-aged population in Poland. A greater importance should be given to potentially modifiable risk factors of dementia which are already present in mid-life.

## Background

Most studies of age-related cognitive changes have focused on neurodegenerative diseases in the elderly. The rationale for this approach is that age is a significant risk factor for cognitive impairment [1]. The most common cause of cognitive deterioration in late life is progressive, irreversible neuronal damage associated with two dominant diseases, Alzheimer’s disease (AD) and dementia due to cerebral small vessel disease (CSVD), i.e. vascular dementia (VaD). For many decades, these have been considered separate diseases with different causes. However, recent findings indicate that markers of CSVD are common in individuals diagnosed with AD [2], and, after accounting for non-independence between risk factors, about one-third of AD cases worldwide might be attributed to potentially modifiable factors [3]. Some of the potentially modifiable factors may depend on lifestyle and clinical status of an individual. Hypertension, diabetes mellitus and smoking have all been reported to be associated with covert infarcts and white matter lesions [4]. However, the effects of diabetes, obesity, diet, alcohol intake and stress have not been well established in population-based studies [5]. Nevertheless, the Alzheimer Association pointed out that good management of cardiovascular risk factors might be associated with reduced risk of dementia [6].

Therefore, it seems that regardless of the clinical classification, cerebrovascular contribution to neurodegenerative diseases will be relevant to the majority of the population living with dementia. So far, no efficient therapeutic solutions have been developed. Moreover, as with other pathological mechanisms in dementia, vascular risk factors can cause brain atrophy years before the onset of symptoms and before the diagnosis [7]. Brain lesions from CSVD may accumulate subclinically over decades; and by the time, they are recognized clinically it may be too late for effective intervention [8]. It is difficult to know how early these lesions may start to affect cognition, due to the scarcity of research into middle-aged cognitive impairment, and little is known about the prevalence of CSVD in people under 65 years of age [9, 10].

Given the above, there seems to be a complex relationship between the vascular risk factors and dementia throughout life. While large epidemiological studies have shown that traditional vascular risk factors are very important determinants of dementia, including AD, the magnitude of their importance appears to be greater in mid-life compared with late life [11–13]. Hence, a detailed risk analysis of people under 65 years of age may identify why this life period is critical for establishing risk for dementia in later life.

Identifying risks in middle-aged people may enable the development of specific guidelines for the prevention of cognitive impairment, facilitating timely intervention in risk groups. As Norton et al. previously indicated, AD incidence might be limited through better access to education and the use of effective strategies to reduce the prevalence of vascular risk factors [3].

To obtain a better picture of risk factors and their influence on cognition in people under 65 years of age, it is necessary to analyse the early symptoms of the emerging neurodegenerative processes. Mild cognitive impairment (MCI) is defined as objective evidence of low cognitive performance in the absence of dementia and is a transition period through which persons pass when they develop dementia [14]. Comprehensive neuropsychological testing can be used to diagnose MCI, but it is not practical for widespread use in primary care. On the other hand, the Folstein Mini Mental Status Exam (MMSE) though practical and widely used is not sensitive for cognitive deficits in MCI and is heavily weighted towards memory and orientation and relatively less sensitive to executive dysfunctions which are prominent in cognitive impairment resulting from covert cerebral ischemia [15]. Thus, in this study, we used other tools of neuropsychological assessment such as Montreal Cognitive Assessment (MoCA) together with the Digit Symbol Substitution Test (DSST) which are more sensitive to executive dysfunction, prominent in cognitive impairment related to CSVD [16, 17].

The aim of the study was to evaluate the prevalence of a wide range of socio-demographic, clinical and health-related factors, as well as CSVD in the middle-aged population and their early impact on cognition assessed using sensitive psychometric tools. In more details, the study aimed to (i) explore prevalent conditions that may contribute to increased risk of MCI during midlife, (ii) explore the contribution of cerebrovascular disease to midlife cognitive impairments and (iii) evaluate the sensitivity of different neuropsychological assessments to cognitive functioning associated with imaging markers of CSVD. In order to unravel complex longitudinal relationships between early exposure to risk factors and clinical outcome, we focused on a cohort of the relatively young people under 65 years of age, which is a unique aspect of this study.

## Methods

### Population

The presented results are part of the National Science Centre grant (NCN nr 2015/17/B/NZ7–02963) called the PURE-MIND study nested within the Polish fraction of the larger multinational PURE study (Prospective Urban Rural Epidemiology Study). The presented PURE-MIND sub-study was a cohort study carried out in 2016–2018, which aimed to explore relationships between socio-demographic and health-related factors, including CSVD and cognition.

**Fig. 1:**
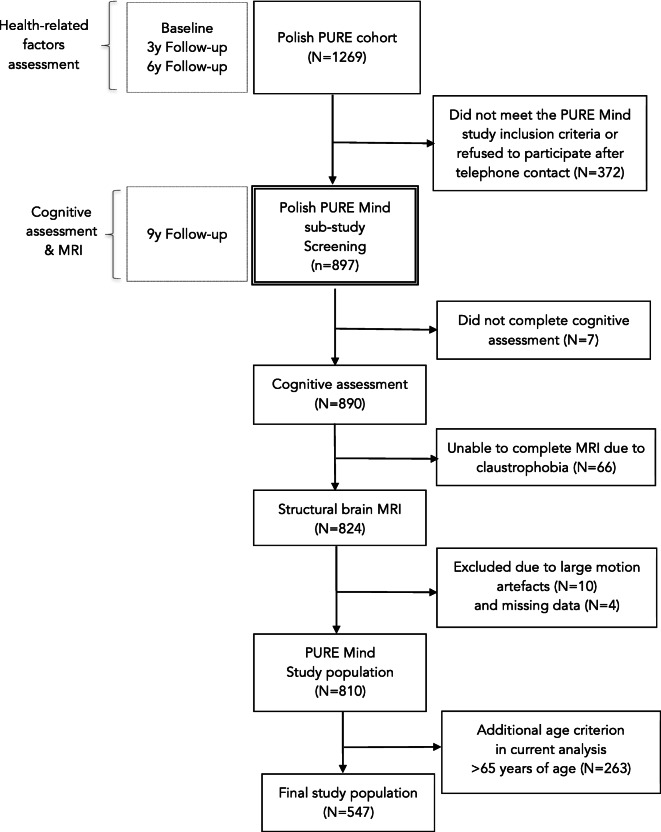
Flowchart of the PURE study participants (*N* = 1269) and the final study population (*N* = 547)

The Pure-MIND study participants were recruited from the PURE study cohort of 1269 people, all of whom were inhabitants of the Lower Silesia region in Poland. The age and place of residence structure of this cohort corresponds to the structure of the general population in Poland. However, the dominance of women is visible in the PURE study sample. A total number of 824 individuals met the eligibility criteria and completed a full study protocol related to a cognitive assessment and underwent magnetic resonance (MR) examination. The PURE-MIND study exclusion criteria were the following: contraindications to MRI (including mainly a pacemaker and other contraindicated body implants, as well as severe claustrophobia), history of stroke or dementia, history of other neurological diseases of the brain, the presence of significant psychiatric diseases, residence in a skilled nursing facility and inability to participate in cognitive assessments (e.g. due to aphasia). Due to large motion artefacts on MRI and missing data, the PURE-MIND study sample consisted of 810 subjects (509 women and 301 men; mean age: 60.9 years; range: 39–81) (response rate 64%). Moreover, an additional criterion of age under 65 years was used in order to focus on middle-aged participants in the current analysis. Therefore, the final study sample consisted of 547 people (195 men and 352 women) with a mean age of 56.2 ± 6.5 years (aged 39–65 years). The flowchart diagram shows separate steps of the recruitment of the final study population (Fig. 1).

All participants received complete information about the study protocol, their anonymity and the possibility to resign at any time. All participants were volunteers and signed an informed consent prior to the study enrolment. The study protocol was approved by the local Bioethical Committee (permission no.: KB – 32/2016).

### Demographic and health-related factor assessment

A comprehensive set of socio-demographic and health-related data was collected as a part of the PURE study and was the basis for a detailed analysis of risk factors of cognitive impairment (Fig. 1.) All participants were examined according to the global PURE study protocol [18] using detailed questionnaires, physical examination and laboratory tests (at baseline, 3-year and 6-year follow-ups). This was followed by neuropsychological assessment and MRI of the brain (at 9-year follow-up) conducted according to the PURE-MIND sub-study protocol.

The baseline socio-demographic and health-related data was collected between years 2007 and 2010 from urban and rural populations of the Lower Silesia region in Poland. The participants were re-contacted every 3 years. Medical information used for this analysis was further collected between years 2007 and 2016 (at baseline, 3-year and 6-year follow-ups) and comprised the presence of hypertension, diabetes mellitus, hyperlipidaemia, coronary heart disease, smoking status, alcohol intake and body mass index (BMI). Clinical characteristics were assigned based on their presence over time from baseline to 3-year or 6-year follow-ups. Longitudinal data collection was used to determine the presence of prevalent conditions (and not the length of their presence) and how those contributed to the cognitive outcome.

Blood pressure measurements were carried out using an automated oscillometric device (Omron Corporation, Tokyo, Japan). The patients were advised to sit quietly and rest for 5 min before the measurements. Appropriate cuff size was selected. Three measurements were taken at 5-min intervals. The average of the measurements was used to diagnose hypertension, according to the ESC criteria (systolic blood pressure 140 or above and/or diastolic blood pressure 90 mmHg or above). Hypertension was considered to be controlled if the objectives of the ESH/ESC Guidelines were met (blood pressure < 140/90 mmHg) [19].

The participants were assigned to the diabetes group when their fasting plasma glucose was ≥ 126 mg/dL (7.0 mmol/L) or when they had been diagnosed with diabetes in the past and had been treated since then. Hyperlipidaemia was diagnosed when the level of total cholesterol was > 190 mg/dL, the level of LDL-C was > 115 mg/dL, the level of HDL-C was <50 mg/dL in women and < 40 mg/dL in men and the triglyceride level was > 150 mg/dL [20].

Coronary heart disease was based on self-reporting of angina, myocardial infarction, coronary artery bypass graft surgery, or percutaneous coronary angioplasty (each category was not separately identified).

Moreover, the smoking status and alcohol intake were assessed.

The body mass of the patients was measured with the use of Tanita Ironman Body Composition Monitor Model BC-554 with accuracy of 0.1 kg. Body mass index (BMI) was calculated as weight (kg) divided by height (m) squared. Subjects were classified into four BMI categories according to the WHO guidelines as being underweight (BMI < 18.5 kg/m^2^), normal weight (BMI 18.5–24.9 kg/m^2^), overweight (BMI 25.0–29.9 kg/m^2^) and obese (BMI ≥ 30.0 kg/m^2^).

### Cognitive assessment

Cognitive evaluation was designed to efficiently assess multiple domains and to minimize the time and participants’ fatigue. It was based on the harmonization standards of the Canadian Stroke Network and the National Institute of Neurological Disorders and Stroke used for the assessment of vascular cognitive impairment recommended in the consensus. The following standardized psychometric tools were used:

The MoCA (The Montreal Cognitive Assessment) scale in Polish adaptation was used to detect cognitive impairment. The published cut-off score (< 26 points) identifies MCI or dementia with high sensitivity and specificity [16]. For univariate analyses, a 1-point adjustment for subjects with less than 12 years of education was used. The MoCA assesses various cognitive domains: attention and concentration, executive functions, memory, language, visuo-constructional skills, conceptual thinking, calculations and orientation. Time to perform the test is about 10 min. The total possible score is 30 points with 26 points or above considered as cognitive health. In present statistical analyses, MoCA was a psychometric tool for measuring general cognitive functioning as a quantitative variable. Moreover, the score below 26 points was defined as mild cognitive impairment (MCI) and was used in the risk factor analysis as qualitative variable. Scores between 26 and 30 points were defined as the cognitive norm.

The DSST (Digit Symbol Substitution Test, Wechsler Adult Intelligence Scale, 3rd Edition) a 2-min test sensitive to covert cerebral ischemia that requires matching a symbol with a number according to the code [17]. The score is equal to the sum of correct symbols drawn in a limited time.

TMT (Trail Making Test) Parts A and B were used to measure cognitive flexibility and central executive functioning. TMT-A version requires combining numbers from 1 to 25, while TMT-B version requires combining numbers and letters alternately [21]. The result is execution time for both versions, number of hints and number of errors in order of digits. Higher scores reveal greater impairment.

The psychological examination was standardized in terms of the procedure and conditions to minimize the dependence of the test scores on the influence of external factors. Each study participant was assessed during an individual contact with a trained psychologist, in a quiet room, while maintaining silent conditions. The instructions were read in the same way each time. The examination was scheduled at a time convenient for the participants.

### Functional assessment

The following tools were used to assess the overall functioning:

CES-D (The Centre for Epidemiological Studies Depression) which is a scale in Polish adaptation to assess depression symptoms in epidemiological studies [22]. Higher scores reveal greater likelihood of depressive symptoms.

SAGE (The Standard Assessment of Global Activities in the Elderly) which is a screening tool to recognize and detect early loss of independence [23]. Higher scores reveal greater impairment.

### Brain MRI acquisition and analysis

All brain MR examinations were performed using the same 1.5 Tesla MR scanner (GE, Signa Hdx). The MRI sequences included axial dual echo T2/PD-weighted images (TR = 2720 ms, TE = 88/8.8 ms, ET [Echo train length] = 12, FOV = 240 × 240 mm, slice thickness = 3.5 mm, matrix = 256 × 256, NEX = 1), axial fluid attenuated inversion recovery sequences (FLAIR) (TR = 8.800 ms, TE = 145 ms, TI = 2.200 ms, FOV = 240 × 240 mm, slice thickness = 3.5 mm, matrix = 256 × 256, NEX = 1), diffusion-weighted imaging (DWI) (SE/EPI, TR = 10,000 ms, TE = 107, FOV = 240 × 240 mm, slice thickness = 3.5 mm, matrix 256 × 256), susceptibility-weighted imaging (SWI) 3D (TR = 73.9 ms, TE = 47.4 ms, FOV = 240 × 168 mm, slice thickness = 3.5 mm, matrix 256 × 256, NEX = 0.7, flip angle = 20) and high-resolution T1-weighted images (FSPGR, TR = 8.3 ms, TE = 3.2 ms, TI = 650 ms, FOV 240 × 240 mm, slice thickness = 1.0 mm, matrix 256 × 256 mm, NEX = 1, flip angle = 12).

All MR scans were evaluated in relation to the findings characteristic of CSVD, such as lacunar infarcts, white matter hyperintensity (WMH) and microbleeds, according to the standard published criteria [10]. Lacunar infarcts were described as small focal lesions up to 15 mm with the same characteristics as cerebrospinal fluid on all sequences located in the subcortical white matter or within deep grey matter structures. White matter hyperintensities were graded on the FLAIR images using the Fazekas scale of 0–3 [24], separately for periventricular and subcortical locations. Burden of WMH was established using cut-off value of 4 for a combined periventricular and subcortical Fazekas grade (low WMH burden was defined as grade 1–3, while high WMH as grade ≥ 4). Cerebral microbleeds were described as small foci of signal loss with blooming effect on SWI.

Severe CSVD was defined as the presence of high WMH burden or lacunar infarcts or microbleeds. Mild CSVD was defined as low WMH burden without lacunar infarcts and microbleeds.

To compare cognitive test scores across lesion types, we grouped participants as follows: healthy (no brain lesions), infarcts (all subjects with lacunar infarcts with or without WMH or microbleeds), high WMH (high WMH burden with or without microbleeds), low WMH (low WMH burden with or without microbleeds) and microbleeds alone.

All MR scans were rated by two independent radiologists trained for CSVD assessment using the same criteria. The inter-rater reliability was established with kappa coefficient that ranged between 0.71 and 0.88 depending on the category of the assessed parameters. Despite the good inter-rater reliability, all scans that showed any discrepancy between the two raters were re-evaluated and the final score was established by consensus.

### Statistical analysis

Differences between the groups of quantitative variables (MRI-related groups and MoCA<26 defined as MCI) were assessed using the Kruskal-Wallis test with Dunn’s non-parametric all-pair comparison and Holm correction or Mann Whitney test when appropriate.

Pearson chi-square test was used to assess differences in the qualitative variables. Correlation between cognitive outcomes and age was calculated using Spearman rank correlation. The optimal cut-off point for age in relation to MoCA below 26 points and 1.5 standard deviation below the mean of DSST score commonly used as clinical thresholds for MCI was determined by highest Youden index in ROC analysis. The analysis of risk factors of cognitive impairment, measured with the MoCA scale (MoCA used as a qualitative variable: < 26 defined as MCI), was calculated by estimation of prevalence ratios using the Poisson regression with robust variance. Moreover, the comparison of the study population to general population was done by comparing proportion confidence intervals of sample proportion to Statistics Poland data. The significance level was set at *p* < 0.05. Analysis was performed using R for windows (version 3.6.1) [25].

## Results

### Study sample

The study sample (*n* = 547) characteristics are shown in Table 1. The majority were women (64%), living in urban areas (74%), with tertiary (41%) or secondary (41%) education. All subjects were characterized as independent after being assessed by SAGE scale (mean score 2.33 ± 2.9, median 1). The average level of depressive symptoms was low (mean score 9.66 ± 8.0, median 8), indicating general lack of depression among the study participants.

**Table 1:** Characteristics of the study population

	Characteristics			Female	Male	*p* value
9 year follow-up	Age	Mean in years (SD)* Range	56.24 (6.5)* [39–65]	56.9 (6.3)*	55.1 (6.8)*	0.003
	Sex, *N* (%)	Female	352 (64)			
		Male	195 (36)			
	Education level, *N* (%)	Primary	27 (5)	16 (4.5)	11 (5.6)	0.4
		Trade school	68 (12)	41 (11.6)	27 (13.8)	
		Secondary/High school	227 (42)	155 (44.1)	72 (36.9)	
		College/University	225 (41)	140 (39.8)	85 (43.7)	
	Living location, *N* (%)	Urban	403 (74)	255 (72.4)	148 (75.9)	0.4
		Rural	144 (26)	97 (27.6)	47 (24.1)	
	Cognitive functioning, *N* (%)	MoCA < 26	178 (33)	117 (33.2)	61 (31.3)	0.7
		MoCA mean score (SD)*	26.29 (2.5)*	26.21 (2.6)*	26.43 (2.5)*	0.3
	Brain pathology in structural MRI, *N* (%)	No brain pathology	150 (27)	79 (22.4)	71 (36.4)	0.002
		Low WMH burden	352 (64)	241 (68.5)	111 (56.9)	
		High WMH burden	15 (3)	12 (3.7)	2 (1.1)	
		Lacunar infarcts	16 (3)	8 (2.3)	8 (4.1)	
		Microbleeds	14 (3)	11 (3.1)	3 (1.5)	
	Depressive symptoms	CES-d mean score (SD)*	9.66 (8.0)*	10.60 (8.7)*	7.97 (6.3)*	0.001
	General activity	SAGE mean score (SD)*	2.33 (2.9)*	2.47 (3.1)*	2.08 (2.4)*	0.4
Baseline, 3 & 6 year follow-up	Hypertension, *N* (%)	No	334 (61)	126 (35.8)	87 (44.6)	0.05
		Yes	213 (39)	226 (64.2)	108 (55.4)	
	Diabetes Mellitus, *N* (%)	No	467 (86)	46 (13.1)	30 (15.4)	0.5
		Yes	76 (14)	306 (86.9)	161 (82.6)	
	Hyperlipidaemia, *N* (%)	No	206 (38)	119 (34.0)	87 (45.3)	0.01
		Yes	336 (62)	231 (66.0)	105 (54.7)	
	Coronary heart disease, *N* (%)	No	511 (93)	330 (93.8)	181 (92.8)	0.8
		Yes	36 (7)	22 (6.2)	14 (7.2)	
	Smoking, *N* (%)	Current	101 (18)	72 (20.5)	29 (14.9)	0.02
		Former	185 (34)	105 (29.8)	80 (41.0)	
		Never	261 (48)	175 (49.7)	86 (44.1)	
	Weight category, *N* (%)	Normal weight	177 (33)	122 (34.7)	55 (28.5)	0.3
		Overweight	149 (27)	135 (38.4)	84 (43.5)	
		Obese	219 (40)	95 (26.9)	54 (28.0)	
	Alcohol intake, *N* (%)	No	136 (25)	106 (30.1)	30 (15.4)	0.0002
		Yes	411 (75)	246 (69.9)	165 (84.6)	

*data expressed in means and standard deviations (SD); Pearson chi-square test was used to assess gender differences in the qualitative variables and Mann-Whitney test to assess gender differences in the quantitative variables; Normal weight (BMI < 25), overweight (BMI 25.0–29.9), obese (BMI ≥30)

The total mean MoCA score was 26.29 ± 2.5 (median 27), and 33% (*n* = 178) of the study participants scored below the recommended cut-off for MCI or dementia (< 26 points). Only 12% (*n* = 67) of the subjects confirmed subjective cognitive impairment (SAGE scale).

In 27% (*n* = 150) of the participants, no brain pathology was detected on MRI, while in 73% (*n* = 397) of the subjects findings related to CSVD were revealed. Mild CSVD characterized by low WMH burden was found in 64%, while severe CSVD was detected in 9% of the participants, including high WMH burden (3%), lacunar infarcts (3%) and microbleeds (3%).

In addition, Table 1 shows the characteristics of the study group in terms of other health-related factors such as hypertension, diabetes mellitus, hyperlipidaemia, coronary heart disease, weight, smoking and alcohol intake. Moreover, gender differences are pointed out in terms of all characteristics. Detailed data are given in cases of significant differences between sexes.

### Cerebral small vessel disease and cognitive functioning

Higher WMH burden was associated with lower MoCA score (rho = −0.17, *p* < 0.00009) and lower DSST score (rho = −0.19, *p* < 0.00001). Moreover, participants with high WMH burden needed more time to perform TMT part A (rho = 0.17, *p* < 0.00008) and part B (rho = 0.21, *p* < 0.00001).

**Fig. 2:**
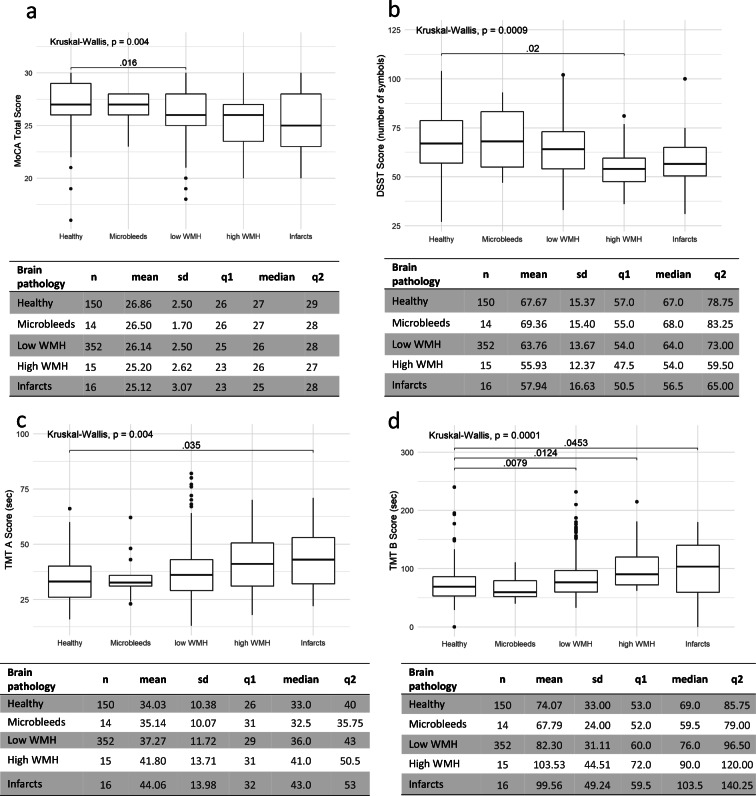
The differences between healthy participants and those with different lesion types in selected cognitive outcomes: (**a**) MoCA, (**b**) DSST, (**c**) TMT A and (**d**) TMT B. Data from participants grouped by lesion types: healthy (no lesions, *n* = 150), infarcts (with or without WMH or microbleeds, *n* = 16), high WMH (with or without microbleeds, n = 15), low WMH (with or without microbleeds, *n* = 352) and microbleeds alone (*n* = 14) are shown as mean and standard deviation (sd). The tables contain information about the median, q1 (quartile 1) and q2 (quartile 2). The charts show statistically significant (*p* < 0.05) differences in cognitive outcomes between healthy participants and the subjects with different cerebral lesions assessed by Kruskal-Wallis test with Dunn’s non-parametric all-pairs comparison and Holm correction

Further analysis showed that participants categorized within 5 groups (healthy, microbleeds, low WMH, high WMH, infarcts) did not show any significant differences in the memory domain based on delayed recall in the MoCA scale (healthy: 3.1 ± 1.6, median: 4; microbleeds: 3.0 ± 1.2, median: 3; low WMH burden: 2.9 ± 1.5, median 3; high WMH burden: 2.5 ± 1.6, median 3; lacunar infarcts: 2.7 ± 1.7, median 3; *p* < 0.2). However, significant differences were observed in the total MoCA score (*p* < 0.004), DSST (*p* < 0.0009) and TMT parts A (*p* < 0.004) and B (*p* < 0.0001) performance (Fig. 2), and the subjects with high WMH or lacunar infarcts scored lower in all these tests. However, significant differences between healthy participants and those with high WMH were observed only in DSST (*p* = 0.02) and TMT B (*p* = 0.0124). In turn, for lacunar infarcts, TMT was the most sensitive psychometric test (part A: *p* = 0.035 and part B: *p* = 0.0453). Moreover, a statistically significant difference between healthy group and a group with a low WMH burden was already shown in the general cognitive functioning outcome (MoCA: *p* = 0.016) as well as in the TMT B (*p* = 0.0079) which stresses central executive processes of task-set inhibition and cognitive flexibility. Participants with microbleeds did not differ significantly from the healthy group.

**Fig. 3:**
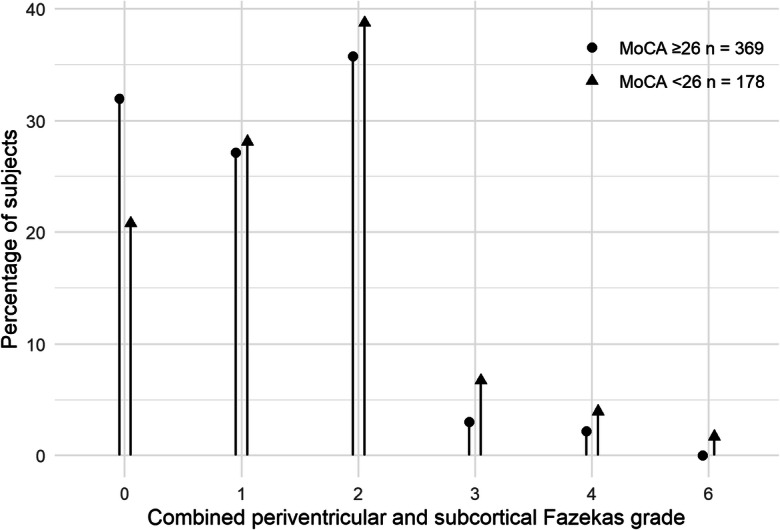
The relationship between white matter hyperintensities (WMH) burden defined as healthy (0), low WMH burden (1–3), high WMH burden (≥ 4) and cognitive performance categorized based on the MoCA score as cognitive health (MoCA ≥ 26, *n* = 369) and cognitive impairment (MoCA < 26, *n* = 178)

In addition, 21% (*n* = 37) of participants meeting the criteria for MCI (< 26 points in MoCA) had normal brain MRI without any CSVD lesions, while 10 participants with high WMH burden had scores corresponding to the cognitive norm in the neuropsychological tests (Fig. 3).

### Sociodemographic as well as health-related factors and cognitive functioning

**Fig. 4:**
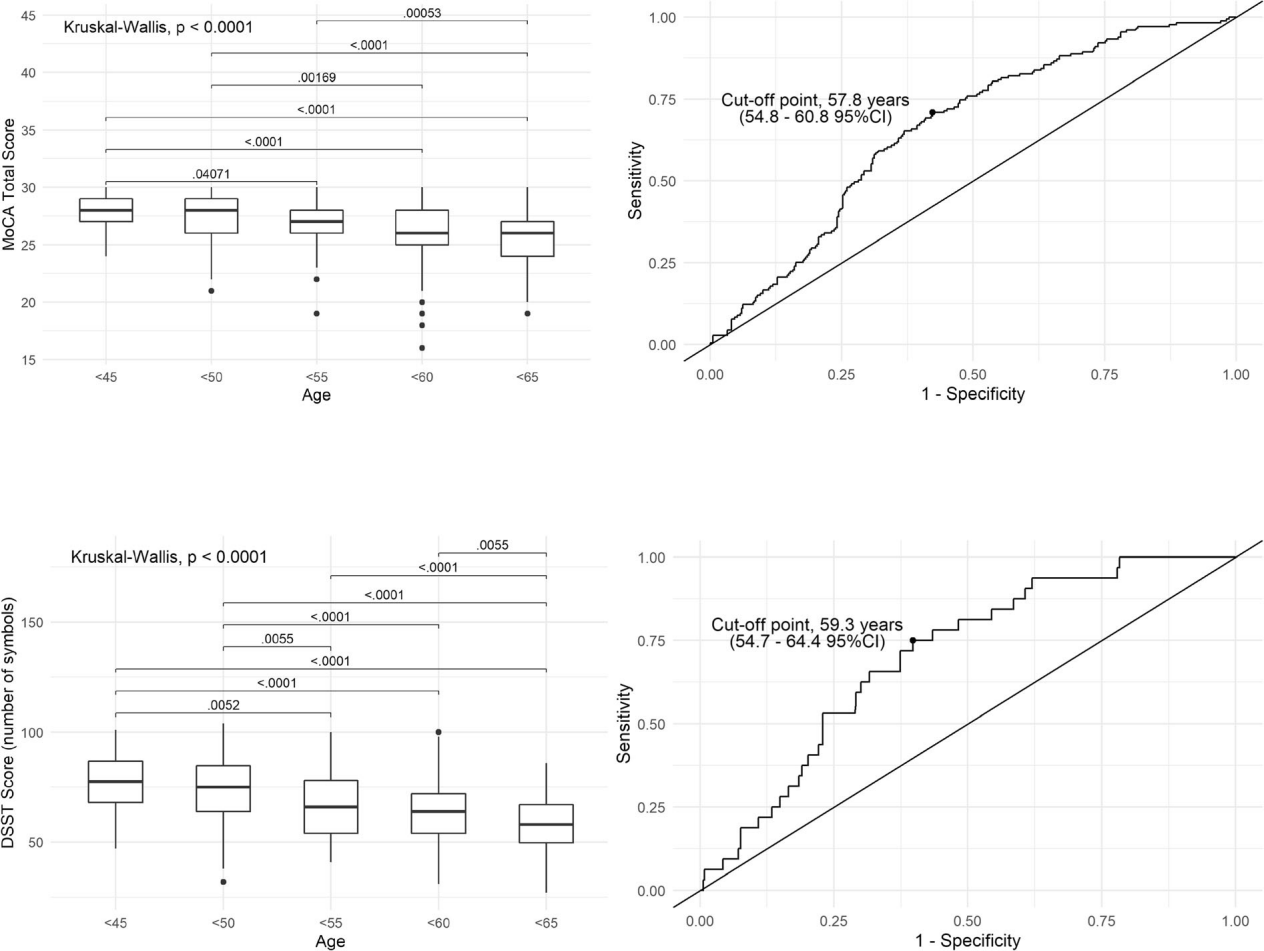
The decline in cognitive functioning with age and receiver operating characteristic (ROC) curves for MoCA and DSST differentiating between cognitive health and cognitive impairment

Table 2 illustrates demographic and health-related variables in the context of cognitive functioning in the middle-aged population. The overall cognitive performance, measured with MoCA (rho = −0.29, *p* < 0.000001), and the ability to learn on new visual material (DSST: rho = −0.42, *p* < 0.000001) decreased with age. Moreover, with age, participants needed more time to complete the TMT A (rho = 0.42, *p* < 0.000001) and TMT B (rho = 0.41, *p* < 0.000001). Figure 4 shows the decline in cognitive function divided by age groups together with the illustration of the ROC curve, which determines the optimal cut-off point in years when this decrease appears to be the most significant. For both MoCA and DSST scales, the relevant cut-off points for age were 58 and 59 years, respectively. There was no significant difference between sexes in terms of general cognitive performance (MoCA: *p* < 0.3). However, speed processing, working memory, visuospatial processing and attention, as well as the ability to learn on new material were higher in women (DSST: *p* < 0.004). However, in terms of executive functions such as processes of task-set inhibition and cognitive flexibility, men showed better scores (TMT A: *p* = 0.02). Moreover, there was a clear effect of education—higher or secondary education level was associated with higher cognitive functioning (MoCA, DSST, TMT A and TMT B, *p* < 0.000001). However, there was no significant difference between people with education at primary and vocational level (*p* < 0.5). Urban residents scored higher on all cognitive tests compared with people living in rural areas.

**Table 2:** Association between sociodemographic, health-related factors and cognitive functions

Characteristics		MoCA		Digit Symbol Substitution		TMT A		TMT B	
		rho	*p* value	rho	*p* value	rho	*p* value	rho	*p* value
Age		−0.29	<0.000001	−0.42	<0.000001	0.42	<0.000001	0.41	<0.000001
		Total mean score (SD)	*p* value	Total mean score (SD)	*p* value	Total mean score (SD)	*p* value	Total mean score (SD)	*p* value
Sex	Female	26.21 (2.6)	0.3	65.90 (14.0)	<0.004	37.18 (11.3)	0.06	82.89 (33.9)	0.02
	Male	26.43 (2.5)		62.22 (14.9)		35.70 (12.2)		76.91 (31.2)	
Education level	Primary	24.12 (2.7)	<0.000001	51.85 (13.5)	<0.000001	44.81 (17.6)	<0.000001	112.70 (44.2)	<0.000001
	Trade school	24.69 (2.7)		52.91 (12.7)		42.25 (13.4)		95.35 (39.5)	
	High school	25.91 (2.5)		62.56 (12.6)		37.47 (10.2)		84.58 (32.1)	
	College/University	27.41 (1.9)		71.70 (12.9)		33.14 (10.2)		68.60 (24.0)	
Living location	Urban	26.63 (2.3)	<0.00001	67.12 (14.2)	<0.00001	35.63 (11.2)	0.0004	78.15 (31.2)	0.0009
	Rural	25.20 (2.7)		57.51 (12.7)		39.49 (12.3)		88.02 (36.8)	
Hypertension	Yes	25.69 (2.7)	<0.00005	59.54 (13.0)	<0.00001	39.97 (12.4)	<0.000001	89.63 (34.9)	<0.000001
	No	26.67 (2.4)		67.81 (14.4)		34.54 (10.6)		75.12 (30.6)	
Diabetes Mellitus	Yes	25.75 (2.7)	<0.05	57.50 (14.0)	<0.000003	41.47 (13.4)	0.0007	93.68 (39.3)	0.00005
	No	26.39 (2.5)		65.75 (14.3)		35.96 (11.1)		78.79 (31.6)	
Hyperlipidaemia	Yes	26.42 (2.4)	0.3	65.04 (13.8)	0.3	36.44 (11.3)	0.8	80.11 (31.4)	0.8
	No	26.11 (2.7)		63.77 (15.7)		37.11 (12.1)		82.09 (35.9)	
Coronary heart disease	Yes	25.92 (2.0)	0.2	58.31 (15.2)	0.02	39.17 (13.5)	0.4	86.22 (31.6)	0.2
	No	26.32 (2.6)		65.03 (14.3)		36.47 (11.4)		80.37 (33.2)	
Smoking	Current	25.94 (2.6)	0.1	60.66 (12.4)	0.0002	38.72 (12.8)	0.2	90.79 (43.4)	0.01
	Former	26.19 (2.5)		63.26 (14.2)		35.94 (19.0)		81.57 (28.7)	
	Never	26.49 (2.5)		67.05 (14.9)		36.36 (12.1)		76.33 (30.6)	
Weight category	Normal weight	26.71 (2.4)	0.007	70.11 (13.3)	<0.000001	34.74 (10.3)	0.03	74.57 (29.1)	0.0004
	Overweight	26.24 (2.5)		63.31 (14.4)		37.22 (12.3)		82.07 (35.1)	
	Obese	25.88 (2.6)		59.85 (13.9)		38.23 (11.8)		86.33 (33.7)	
Alcohol intake	Yes	26.42 (2.5)	0.03	65.29 (14.0)	0.06	35.99 (11.1)	0.07	79.32 (31.8)	0.1
	No	25.88 (2.7)		62.47 (15.5)		38.64 (12.9)		85.08 (36.4)	

Kruskal-Wallis rank sum test or Mann-Whitney test were user to assess differences in the quantitative variables; Correlation between cognitive outcomes and age was calculated using Spearman rank correlation; Normal weight (BMI < 25), overweight (BMI 25.0–29.9), obese (BMI ≥ 30)

Among health-related variables, hyperlipidaemia was not significantly associated with cognitive functioning (MoCA and DSST, *p* < 0.3; TMT A and B, *p* = 0.8). Other risk factors showed differences in the influence on cognition depending on the measured domains. MoCA as well as TMT A results showed that coronary heart disease and smoking were not related to the global cognitive functioning. However, the DSST task (more related to learning processes) was related to these factors (*p* < 0.02 and *p* < 0.0002, respectively). The results indicated that the participants who had never smoked showed significantly higher scores compared with the current (*p* < 0.0009) and former (*p* < 0.005) smokers. Participants who indicated abstinence from alcohol had lower scores on all tests, but this was only significant for the MoCA (*p* < 0.03). Hypertension, diabetes and obesity were associated with lower scores on all tests.

### Mild cognitive impairment risk factors

Analysing variables independently of each other (Tab. 3) indicated that risk factors for decreased cognitive functioning which met the MCI criteria assessed by MoCA (< 26) were older age (*p* < 0.000001), rural area of residence (*p* < 0.000003) and hypertension (*p* < 0.0002). Participants with such characteristics significantly more often scored below 26 points in MoCA. On the other hand, significant protective factors were higher education level (*p* < 0.000001), normal body weight (*p* < 0.003) and alcohol intake (*p* < 0.05).

**Table 3:** Association between sociodemographic, health-related factors and mild cognitive impairment versus cognitive health

Characteristics		MoCA <26	MoCA >26	*p* value
Age	Mean in years (SD)	58.77 (5.2)	55.02 (6.7)	<0.000001
		*N* (%)		
Sex	Female	117 (33.2)	235 (66.8)	0.7
	Male	61 (31.3)	134 (68.7)	
Education level	Primary	18 (66.7)	9 (33.3)	<0.000001
	Trade school	42 (61.8)	26 (38.2)	
	High school	88 (38.8)	139 (61.2)	
	College/University	30 (13.3)	195 (86.7)	
Living location	Urban	108 (26.8)	295 (73.2)	0.000003
	Rural	70 (48.6)	74 (51.4)	
Hypertension	Yes	90 (42.3)	123 (57.7)	0.0002
	No	88 (26.3)	246 (73.7)	
Diabetes Mellitus	Yes	27 (35.5)	49 (64.5)	0.6
	No	148 (31.7)	319 (68.3)	
Hyperlipidaemia	Yes	103 (30.7)	233 (69.3)	0.3
	No	72 (34.9)	134 (65.1)	
Coronary heart disease	Yes	14 (38.9)	22 (61.1)	0.5
	No	164 (31.9)	347 (68.1)	
Smoking	Current	40 (39.6)	61 (60.4)	0.07
	Former	65 (35.1)	120 (64.9)	
	Never	73 (27.9)	188 (72.1)	
Weight category	Normal weight	41 (23.2)	136 (76.8)	0.003
	Overweight	75 (34.2)	144 (65.8)	
	Obese	60 (40.3)	89 (59.7)	
Alcohol intake	Yes	124 (30.2)	287 (69.8)	0.05
	No	54 (60.3)	82 (39.7)	

Wilcoxon rank sum test was used to assess differences in age; Pearson chi-square test was used to assess differences in the qualitative variables; Normal weight (BMI < 25), overweight (BMI 25.0–29.9), obese (BMI ≥ 30)

**Fig. 5:**
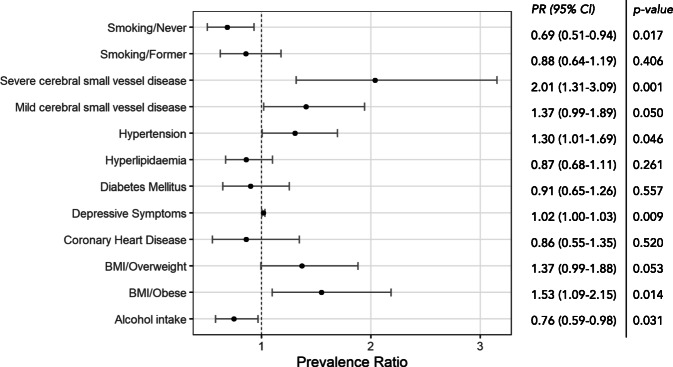
Prevalence ratio (PR) of cognitive impairment (MoCA< 26) with regard to selected risk factors calculated by the Poisson regression with robust variance

Robust Poisson regression model was used for multivariable analysis (Fig. 5). Severe CSVD was associated with twofold higher incidence of MCI (PR 2.01; 95% CI: 1.31–3.09) and mild CSVD was associated with increased prevalence of MCI by 37% (RR 1.37; 95% CI: 0.99–1.89). Overweight was associated with higher rates of cognitive impairments (PR 1.37; 95% CI: 0.99–1.88), and obesity increased the probability of cognitive impairment by 53% (PR 1.53; 95% CI: 1.09–2.15). Among the participants who had never smoked, the likelihood of MCI was reduced by 31% (PR 0.69; 95% CI: 0.51–0.94). Participants who reported consuming alcohol were less likely to have cognitive deficits (PR 0.76; 95% CI: 0.59–0.98). Furthermore, hypertension was associated with the increased risk of cognitive impairment (PR 1.30; 95% CI: 1.01–1.69). A marginal but significant association was found with depressive symptoms (PR 1.02; 95% CI: 1.00–1.03), while no relationship was found with hyperlipidaemia, diabetes and coronary heart disease.

## Discussion

In one third of the community-representative study, including participants aged between 39 and 65 years, MCI range cognitive impairment was detected during the neuropsychological examinations. Only 12% of the participants were classified as having subjective deficits, which means that more than half of the study representatives with objective cognitive impairment were unaware of early signs of cognitive decline. Recent studies of MCI showed a prevalence of 15–20% in people aged 65 years or older [26]. Very limited data are available regarding people below 65 years of age. Other population-based studies included either mainly older adults or did not report specific findings in younger participants. However, the prevalence of cognitive impairment in this study population should be approached with some caution, given that situational factors such as fear of the psychological examination could worsen the results in the cognitive tests, despite maintaining all needed standardized conditions.

Magnetic resonance studies showed that over 73% of the middle-aged participants had CSVD, of which 9% had severe CSVD, defined as high WMH burden, lacunar infarcts or microbleeds. These results are in line with the conclusions of Smith et al. [27] who emphasized that CSVD was detected in a significant proportion of people in their 40s and 50s and that the brain changes were not silent even in relatively young individuals. What is more, Han et al. [28] in a cohort of 1211 stroke-free participants (55.6 ± 9.3 years) also showed the prevalence of 14% for lacunes, 72% for periventricular WMH, 65% for subcortical WMH and 10% for cerebral microbleeds, all of which increased with age. In the current study, higher WMH burden was associated with greater deficits in cognitive functions, and severe CSVD was one of the major risk factors for MCI increasing its incidence by 200%. Learning ability assessed with DSST, speed processing, cognitive flexibility and executive functions measured with TMT A and B tests were also significantly related to high WMH burden and lacunar infarcts. Nevertheless, it should be pointed out that in the neuropsychological assessment, TMT B seems to be of particular importance. It stresses central executive processes of task-set inhibition and cognitive flexibility and showed sensitivity in distinguishing between healthy people and people with all selected lesion types, even in those with low WMH burden. Interestingly, Smith et al. did not find significant associations between WMH and MoCA in the Canadian cohort, which they considered to be the result of too low sensitivity of the MoCA scale to the presence of covert cerebrovascular disease in a population-based setting [27]. However, a recent larger Canadian study demonstrated that associations between WMH and MoCA were present with sufficient sample size, concordant with the findings of this study [29]. Detailed analysis showed that participants without brain pathology on MRI and people with the presence of CSVD differed in all tasks measuring executive functions and cognitive flexibility in the MoCA scale, except for delayed recall. Due to the above, the first signs of morphological brain pathology might be visible primarily in the non-memory cognitive domains. Importantly, this relationship is observed in relatively young people who are expected to have “normal” cognitive productivity. It should also be emphasized that 21% of participants with MCI according to the MoCA test showed no brain pathology in MRI, which may be explained by a non-vascular origin of the symptoms, probably due to a purely degenerative process. On the other hand, 10 subjects with high burden of WMH showed normal results in the psychological tests, which may indicate their good compensatory abilities or cognitive reserve making the early symptoms of cognitive impairment not detectable using the performed tests.

Despite the fact that current analysis included people younger than 65 years of age, overall cognitive performance and learning ability on new material, reflected by performance on the DSST, as well as the speed processing (TMT) was found to decrease with age. The age-related decline in cognitive performance is consistent with findings among the middle-aged London civil servants, as well as many other studies conducted among older population [30]. Moreover, findings from the present study imply the clinically important suggestion that a significant decrease in cognitive functioning is visible already at age of 58 years, while clinical experience shows that in daily practice, specialists begin to deal with cognitive health usually in people around the age of 65 years or older.

The ambiguous conclusions relate to the gender effect in terms of the cognitive outcomes and CSVD. Mild cognitive impairment as well as general cognitive functioning is not related to gender, which is supported by the results of the MoCA test. However, our findings highlight the differences in terms of selected cognitive domain performance between women and men in the study sample. The results show better learning capacity (DSST) in women and better cognitive flexibility (TMT B) in men. Interestingly, the findings show a lower incidence of CSVD in men that could be seen as unexpected result as typically men are more at risk of cardiovascular disease, also in the present sample. Contradictory conclusions are presented by authors of the study from the United Kingdom who found that men were more prone to CSVD [31]. Male sex was a significant and independently associated factor for total CSVD score in that study. However, it should be emphasized that in the present study, women more often than men were classified as having low WMH and high WMH burden, but in men more often than in women lacunar infarcts were detected. Nevertheless, a larger proportion of men compared with women were characterized by a lack of pathological changes in the brain. An important observation that may be related to the data obtained is the gender difference present in depressive symptoms highlighting significantly higher scores on the depression scale in women in the study sample. This observation seems to be of great importance having regard to the significant relationship between depression and WMHs and cerebral atrophy as concluded in the systematic review and meta-analysis conducted by Rensma et al. [32]. Thus, gender differences in CSVD can be understood as a derivative of gender differences in terms of depressive symptoms in this particular study population. However, further analysis is necessary.

In this study, education level was found to be one of the most fundamental protective factors, and participants with higher education performed better in the cognitive assessment. The area of residence also turned out to be significant. In our study, urban living was associated with the better performance in the neuropsychological tests, as confirmed by some studies [33], while contradicting some other reports [34]. There is a likely relationship between education and the area of residence. In Poland, there are still disparities in access to education depending on where you live. Living in the city offers many different forms of cultural and educational participation, which may increase cognitive reserve phenomenon. This indicates the importance of lifestyle factors affecting the cognitive functioning among middle-aged people. It is consistent with Norton’s statement that many dementia cases worldwide might be attributable to potentially modifiable factors [3]. In this study, we found several risk factors for cognitive impairment meeting the MCI criteria that might be modifiable. One of them is obesity, found in our study to increase the probability of cognitive impairment by about 53%, which confirms recent findings on dementia among older adults [35]. Another important clinical risk factor in this study was hypertension, which disrupts the structure and function of cerebral blood vessels and may lead to ischemic damage of the white matter regions critical for cognitive function. Several reports emphasized the deleterious impact of mid-life hypertension on late-life cognitive decline [36, 37]. However, current findings have proved this harmful effect already in the middle-aged participants.

The obtained results confirmed the protective effect of not smoking. The likelihood of MCI was significantly reduced (by about 31%) in people who never smoked. This phenomenon may be related to the fact that these people generally lead a healthier lifestyle, a hypothesis that requires more in-depth examination. Our analysis found that alcohol drinkers were less likely to exhibit cognitive deficits compared with abstainers. This finding is consistent with the previously reported U-shaped relationship between regular alcohol consumption and cognitive function [38]. However, special care should be taken when formulating policy implications on this topic. Moreover, account should be taken of the fact that in this study assessment of alcohol consumption was carried out using interviews. The hard drinkers could deny drinking during interview, whereas people drinking occasionally could more openly confirm drinking alcohol. What is more, the reasons for not drinking alcohol have not been assessed. Which in the light of the results obtained may be of key importance assuming that non-drinkers make such a decision for health reasons and therefore remain in a much worse psychophysical health. In addition, hyperlipidaemia, diabetes and coronary heart disease were not associated with the occurrence of MCI. Nevertheless, there was a relationship found between diabetes and general cognitive performance. Participants who did not have diabetes scored significantly higher on the MoCA.

This study has several strengths. First, we focused on middle-aged participants, who are still an under-studied group. Second, this is the first large-scale study of this kind in Poland. Third, good quality data were obtained through the use of a research MRI with a strict protocol for the assessment of cerebrovascular pathology and a sensitive set of psychometric tools that were used to assess cognitive functioning. Another very important aspect was an access to longitudinal data on a wide range of lifestyle and clinical factors which are more powered than cross-sectional studies. Finally, due to the similarities in age structure and place of residence of the studied cohort at the baseline compared with the general population of Poland, these findings can be extrapolated to the Polish population, nevertheless with some caution. Discrepancies in the population structure such as women’s dominance in the PURE-MIND sub-study sample could result from the exclusion criteria, as well as the greater activity of female volunteers in a scientific research.

On the other hand, the biggest limitation of this study was the relatively small sample size as for a cohort study, since the participants were recruited from only one region in Poland. Moreover, the role of genetics was not analysed in the present study and the importance of genetic factors in the development of dementia and cerebrovascular pathology should not be overlooked. Heritability studies, including twin studies, show that most of the variance in a white matter hyperintensity volume is explained by genetic factors [39, 40]. Nevertheless, the role of the main genetic determinant of AD, apolipoprotein ɛ4 allele (APOE4), is still unclear in vascular cognitive deficits. A study from Singapore emphasized that APOE4 is significantly associated with dementia and cognitive deficits related to AD pathology, but not with vascular dementia [41]. According to Clark et al. [42], APOE genotype did not moderate relationship between risk factors and cerebral perfusion. Thus, despite the important role of genetic factors, the authors suggested that there is a great need to continue exploration of the relationship between potentially modifiable risk factors, cerebrovascular health and AD risk in underrepresented populations.

## Conclusions

In this community-based study conducted on the Polish rural and urban population of people aged between 39 and 65 years, a relatively high prevalence of cognitive impairment was observed. The major clinical risk factors of MCI that already appeared in the middle-life were found to be severe CSVD, obesity and hypertension. Moreover, socio-demographic factors such as place of residence, age and education, as well as lifestyle factors, such as smoking also played an important role in cognitive impairment. In addition, it turned out that people with diabetes achieved significantly lower results in the neuropsychological tests, indicating the importance of this disease for cognitive performance, although it was not ultimately identified as a risk factor for MCI. This probably means that diabetes reduces global cognition, but in people up to 65 years of age, there are no clinically relevant consequences visible in cognitive impairment. Moreover, our findings indicate that among some study participants, the phenomenon of cognitive reserve is observed recognized by proper cognitive functioning despite serious morphological pathologies visible in MRI. Therefore, subsequent research should focus mainly on potential protective factors that improve compensatory possibilities and cognitive reserve of an individual person.

In the light of these findings emphasizing the important influence of lifestyle factors on cognition, improving access to higher education and applying effective strategies targeted at reducing the prevalence of vascular risk factors should become a major focus of health providers and policy makers. However, further research and data analyses are necessary to determine whether such preventive actions will really translate into a reduction in mid-life cognitive impairment and dementia at the later stages of life. Dissemination of knowledge that the first signs of cognitive impairment may be associated with domains other than memory, and information that cognitive impairment is visible before the age of 60 years, needs to be considered in everyday clinical practice.
